# Resistance exercise affects catheter-related thrombosis in rats through miR-92a-3p, oxidative stress and the MAPK/NF-κB pathway

**DOI:** 10.1186/s12872-021-02233-w

**Published:** 2021-09-16

**Authors:** Cui Wen, Yanping Ying, Huihan Zhao, Qingjuan Jiang, Xiao Gan, Yan Wei, Jiani Wei, Xinxin Huang

**Affiliations:** Department of Nursing, The First Affiliated Hospital of Guangxi Medical University, No. 6, Shuangyong Road, Nanning, 530021 Guangxi China

**Keywords:** Catheter-related thrombosis, Resistance exercise intervention, miR-92A-3P, Oxidative stress, MAPK/NF-κB pathway

## Abstract

**Background:**

MiR-92a-3p and oxidative stress are associated with catheter-related thrombosis (CRT). As a kind of physical intervention, resistance exercise can effectively promote blood circulation. In this study, we investigated the roles of miR-92a-3p, oxidative stress and the P38 mitogen-activated protein kinase/nuclear factor-κB (MAPK/NF-κB) pathway in CRT during resistance exercise.

**Methods:**

The rat CRT model was used for resistance exercise intervention. Moreover, pathological changes from the right jugular vein to the right auricle were observed under an electron microscope. In addition, reactive oxygen species (ROS) production, malondialdehyde (MDA) activity and heme oxygenase (HO-1) level in rat serum were detected via ELISA. The expression levels of miR-92A-3p and HO-1 in the vascular tissues of the rats were determined via real-time quantitative PCR. Additionally, the expression levels of HO-1, NF-κB P65, p38MAPK and IκBa in the venous tissues of the rats were analysed by Western blot analysis.

**Results:**

The pathological results showed that the thrombosis incidence rate in the CRT + RE group was lower than that in the CRT group. In the CRT group, the expression levels of ROS and MDA, which are markers related to oxidative stress in serum, significantly increased whilst the expression of HO-1 decreased. In the venous tissue, the expression of miR-92a-3p increased, the level of HO-1 decreased, the levels of p38MAPK and NF-κB p65 significantly increased but that of P-IκBa and IκBa significantly decreased. In the CRT + RE group, after administering the resistance exercise intervention, ROS production and MDA activity in serum significantly decreased, the expression level of HO-1 increased and the expression level of miR-92a-3p in the venous tissues significantly decreased and was negatively correlated with that of HO-1. The levels of p38MAPK and NF-κB p65 significantly decreased but that of P- IκBa and IκBa significantly increased.

**Conclusion:**

Resistance exercise intervention downregulated miR-92a-3p expression, repaired oxidative stress injury and prevented CRT formation.

**Supplementary Information:**

The online version contains supplementary material available at 10.1186/s12872-021-02233-w.

## Introduction

Central venous catheters (CVCs) are extensively applied in medical and health institutions. CVCs can offer safe and reliable venous access to tumour chemotherapy, parenteral nutrition, long-term infusion and treatment of critically ill patients [1]. After intravenous catheterisation, the catheter punctures the vascular wall, resulting in endothelial cell injury and hemodynamic changes and further promoting blood hypercoagulability [2]. Hence, catheter-related complications are inevitable, amongst which catheter-related thrombosis (CRT) is the most severe, resulting in pulmonary embolism, recurrent deep vein thrombosis (DVT), post-thrombotic syndrome and sepsis [3]. Recent studies noted that 16–18% of patients with CVCs have evidence of CRT as revealed by ultrasound or intravenous screening [4]. Moreover, the incidence of asymptomatic CRT is reportedly as high as 68% [5]. CRT cannot be detected without ultrasonic evaluation and testing, and compounding this problem is the fact that only 1–5% of patients are symptomatic [6]. Therefore, CRT can be characterised as a ‘high risk, common’ disease. CRT can increase pain sensitivity, leading to catheter dysfunction, increased risks of infection and central venous stenosis; moreover, it increases the length of hospital stay and medical costs [7]. Therefore, CRT prevention is necessary.

The Infusion Nurse Society, following the amendments of the Infusion Practice Standard of 2016, has encouraged patients undergoing infusion to engage in physical activity and exercise as early as possible to prevent CRT formation [8]. Colwell et al. [9] found no statistically significant differences between anti-coagulant drugs and physical exercise in terms of reducing DVT incidence. Compared with anti-coagulant drugs, physical activity can effectively minimise or eliminate the risk of bleeding. Aerobic exercise and resistance exercise on the side of catheterisation can benefit the increase in blood flow velocity of the heart and blood vessels [10]. Early resistance exercises can increase muscle strength and enhance subendocardial blood perfusion by increasing cardiac pressure load to achieve the optimal balance between cardiovascular oxygen supply and demand and improve cardiovascular functions [11].

MiRNAs are a class of endogenous non-coding small RNA molecules that play an important regulatory role in angiogenesis [12]. Members of the miR-17-92 cluster have a considerable influence on the cardiovascular system. Specifically, miR-92a regulates vascular dynamic balance by upregulating the expression of endothelial pro-inflammatory factors [13, 14]. Micro-92a-3p is reported to be involved in regulating vascular system dysfunction and mediating blood flow shear stress, and its expression is up-regulated in a rat model of DVT [15, 16].

When the vessel wall is punctured by a catheter, oxidative stress damages vascular endothelial cells, leading to ROS and reactive nitrogen over-production and eventually disrupting the redox balance [17]. A high ROS content can increase the level of malondialdehyde (MDA), further causing oxidative stress-induced tissue damage, increasing membrane permeability and rupturing the double-layered structure of the cell membrane [18]. HO-1 (HMOX1), an antioxidant enzyme found in vascular endothelial cells and smooth muscle cells, can protect damaged blood vessels via inhibiting the proliferation of vascular smooth muscle cells [19].

Pro-apoptotic pathways (i.e. mitogen-activated protein kinases [MAPKs]) and inflammatory response pathways (i.e. NF-κB) are important in regulating apoptosis and tissue damage. A sustained increase in ROS can promote endothelial cell apoptosis and activate inflammatory responses through the activation of the MAPK and NF-κB signalling pathways [20]. In vivo experiments have shown that MAPK regulates Ace2 mRNA expression in the aortic vascular smooth muscle cells of rats, indicating that MAPKs may play a role in repairing vascular endothelial injury [21]. However, the signalling pathway that initiates CRT-induced oxidative stress injury in rats remains unclear. Gan's research [22] shows that HO-1 may be the target of miR-92a-3p and p38 MAPK/NF-κB pathway. miR-92a-3p may regulate HO-1/p38 MAPK/NF-κB pathway and result in CRT. Oxidative stress may activate MAPK/NF-κB, mediate endothelial cell apoptosis, promote the expression of tissue factor and platelet secretion, and regulate venous thrombosis.

Resistance exercise is a form of intervention that has been repeatedly validated to reduce the risk of cardiovascular diseases [23]. However, the underlying molecular mechanism by which resistance exercises regulate CRT occurrence has not been elucidated yet. This study investigated the effect of resistance exercise on CRT-induced thrombosis formation. Mechanistically, resistance exercise mitigated oxidative stress and inflammation via regulating miR-92a-3p expression and the MAPK/NF-κB pathway.

## Materials and methods

### Animals

Fifty male Sprague–Dawley (SD) rats weighing 190–240 g were purchased from the Experimental Animal Centre of Guangxi Medical University (Experimental Animal Breeding License Number SCXK Gui 2014-0002). The rats were exposed to 12/12 light/dark cycles in a strictly controlled environment at 21–23 °C and 50–60% humidity. As recommended by the Specific Pathogen Free (SPF) barrier environmental conditions, food, bedding, drinking water and cage devices were thoroughly sterilised. Feeding, disposal of dead experimental animals and access of the experimenters to the animals were meticulously performed according to SPF laboratory regulations.

This study was conducted in accordance with the Guidelines for Ethical Review of Experimental Animals for Animal Welfare in China and was approved by the Animal Protection and Welfare Committee of Guangxi Medical University. This protocol faithfully complied with the Guidelines for Laboratory Animal Care and Use of the National Institutes of Health (NIH Publication No. 85-23). The study was carried out in compliance with the ARRIVE guidelines.

### Grouping and establishment of CRT models

**Fig. 1:**
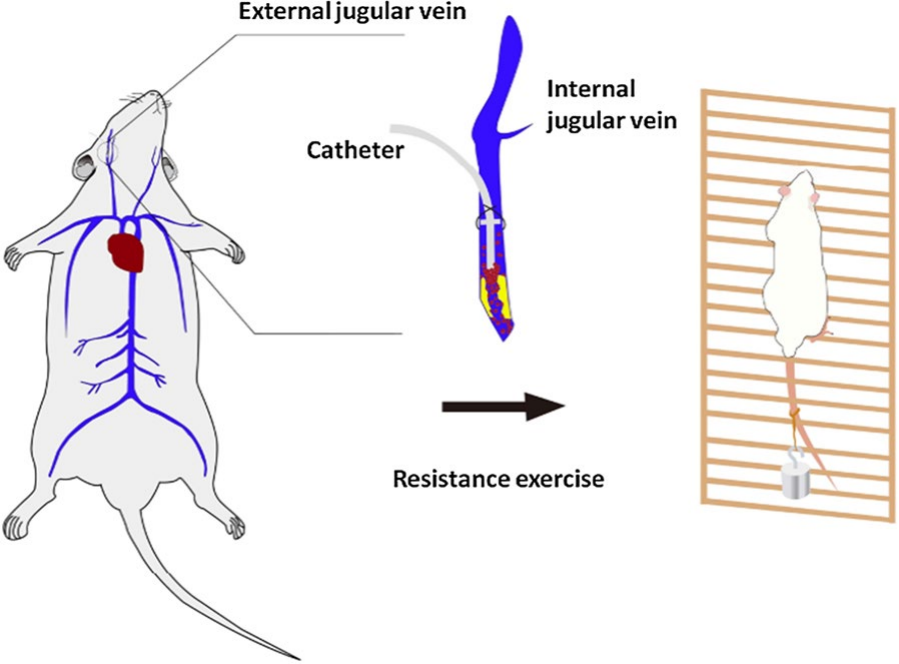
A catheter was inserted into the external jugular vein of the rat. The joint of the catheter was sutured to secure the catheter in proper place. The vascular catheter was fixed to the subcutaneous tissue with sutures, and resistance exercise training began on the 2nd day after the operation

The SD rats were randomly divided into five groups (10 rats in each group) by using a random number generator as follows: control, sham operation (Sham), sham operation + resistance exercise (Sham + RE), CRT and CRT + resistance exercise (CRT + RE) groups. The rats were intraperitoneally injected with 1.5 mL/kg 3% sodium pentobarbital solution under general anaesthesia. The skin of the right neck was shaved and disinfected with iodophor. All surgical instruments were steam-sterilised, and a disposable sterile orifice plate was used to establish a sterile area around the neck. The catheterisation method described by Smith et al. [24] was modified to construct CRT models of the SD rats. Surgery was not performed in the rats in the control group. The right external jugular vein of each rat in the Sham and Sham + RE groups was separated after the neck skin was cut open, into which a 3–3.5 cm-long catheter was inserted to destroy the vascular endothelium. The catheter was quickly pulled out, and the wound was immediately sutured to stop the bleeding. Each rat in the CRT and CRT + RE groups was incised with blunt dissection of the neck skin and the surrounding connective tissues. An oblique incision was made in the right external jugular vein, and a catheter 3–3.5 cm in length was inserted until it reached the superior vena cava (Fig. 1). The syringe connected to the end of the catheter was pumped. After blood reflux and smooth injection with 0.5 mL of normal saline, the catheter near the heart was ligated and fixed. The tube was sealed with a special plug and placed under the skin, and the subcutaneous tissues and skins were sutured. The rats were kept warm and observed until they recovered from the effects of anaesthesia after the operation. Afterward, the rats were fed normally.

### Resistance exercise intervention in rats

All rats underwent 1 week of adaptive training before surgery without weight crawling. The rats that completed six times of crawling per day were included in the experiment; otherwise, they were excluded. The rats in the Sham + RE and CRT + RE groups started resistance exercise on the first day after the operation. The rats were trained in climbing a ladder with a gradient of 85° by attaching heavy weights to the tail [25]. The rats were induced to climb from the bottom of the ladder to the top; they were trained 5 days a week and rested for 2 days. Two groups were trained three times daily per group for 2 min each time. The rats were allowed to rest at the top of the ladder for 20 s each time. The training was conducted for 8 weeks. The initial weight bearing was 10% of the rats’ body weight, and it was gradually increased every succeeding week. In the first, second, third and fourth weeks, the weight bearing was equivalent to 10%, 20%, 30% and 40% of the rats’ body weight, respectively. The weight bearing was increased to 70% of the rat’s body weight from the fifth week to the eighth week and then maintained thereafter [26]. The tails of the rats were stimulated to promote exercise as necessary (Fig. 1).

### Histological observation

Eight weeks later, rats were euthanised with an overdose of pentobarbital, and the right external jugular veins were removed and fixed overnight with 10% neutral buffer formalin. After soaking and fixation, the catheters in the CRT and CRT + RE groups were slowly removed from the vein before dehydration. Paraffin-embedded tissues were cut into five cross-sections. All tissue sections were stained with haematoxylin and eosin, and thrombosis was inspected by pathologists. Images of the stained sections sealed with neutral gum were digitised by using a microscope (BX53, Olympus, Japan). The images were captured using the digital management software cellSens Standard. Two pathologists viewed and rated the images independently.

### ELISA

Serum was isolated from each rat and stored at − 80 °C for ELISA. ROS ELISA kit (ML0262881, Mlbio, Shanghai, China), MDA ELISA kit (ML022446, Mlbio, Shanghai, China) and HO-1 ELISA kit (ML003108, Mlbio, Shanghai, China) were used to detect the corresponding molecule expression levels in the serum. ELISA experiments were performed following the manufacturer’s instructions.

### Quantitative polymerase chain reaction

Total RNA was extracted and purified from 1 cm-thick vascular tissues (Ambition, Carlsbad, USA) by using a Trizol reagent homogeniser. MiRNAs were extracted using miRcute miRNA extraction and separation kits following the manufacturers’ protocols (DP501, Tiangen Biotech). Total RNA was reverse transcribed into cDNA using a miRcute-enhanced miRNA cDNA first-strand synthesis kit (KR211-02, Tiangen Biotech). RT-PCR analysis was performed using a miRcute-enhanced miRNA quantitative fluorescence kit and SYBR qPCR Mix (Monad, Wuhan, China). The primers used for qPCR are presented in Table 1. mRNA expression levels were quantified using the 2^−∆∆Cq^ method and normalised to the internal reference gene β-actin or U6.

**Table 1:** Primer sequences for qPCR

Gene	Forward primer (5′-3′)	Reverse prime (5′-3′)	Product size	Gene bank accession number
U6	CTCGCTTCGGCAGCACA	AACGCTTCACGAATTTGCGT	50 bp	NR_138085.1
miR-92a-3p	ATAACGTGAACAGGGCCG	CAGTGCGTGTCGTGGAGT	50 bp	NR_032335.1
β-actin	CGTAAAGACCTCTATGCCAACA	TAGGAGCCAGGGCAGTAATC	100 bp	NM_031144.3
HO-1	CAGAAGAGGCTAAGACCGCC	GGGGCCAACACTGCATTTAC	288 bp	NM_012580.2

### Western blot analysis

Total protein was extracted from venous tissues of rats in each group lysed with RIPA lysis buffer. Protein concentration was determined via the BCA method (Solarbio, PC0020). Proteins were separated via SDS-PAGE and transferred onto PVDF membranes. The membranes were incubated at 4 °C overnight with the following primary antibodies in 5% BSA: anti-NF-κb P65 (Phosphoo S536 and AB86299, 1:2000; Abcam), anti-NF-κB P65 (AB16502, 1:2000; Abcam), recombinant anti-IκB alpha (E130) (AB32518, 1:1000; Abcam), P38 MAPK (D13E1) XP® Rabbit mAb (CST 8690, 1:1000), phospho-p38 MAPK (Thr180/Tyr182) (12F8) Rabbit mAb (CST 4631, 1:1000), anti-Ho-1 (K002131P, 1:1000; Solarbio) and anti-β actin (AB8227, 1:500; Abcam) antibodies. The blots were washed three times with TBST and then incubated with a secondary antibody (goat anti-rabbit: ab6721, 1:4000; Abcam) for 1 h. An ECL reagent was used to render the membrane, and exposure imaging was performed in a gel imager.

### Statistical analysis

Data were analysed by SPSS 23.0. Data were presented as the mean ± standard deviation. Differences amongst groups were analysed by one-way ANOVA with Tukey’s post hoc test, and rates amongst groups were compared by chi-square test. Pearson correlation analysis was performed using GraphPad Prism 8.3. P < 0.05 was considered statistically significant.

## Results

### Effects of resistance exercise on rat body weight

**Fig. 2:**
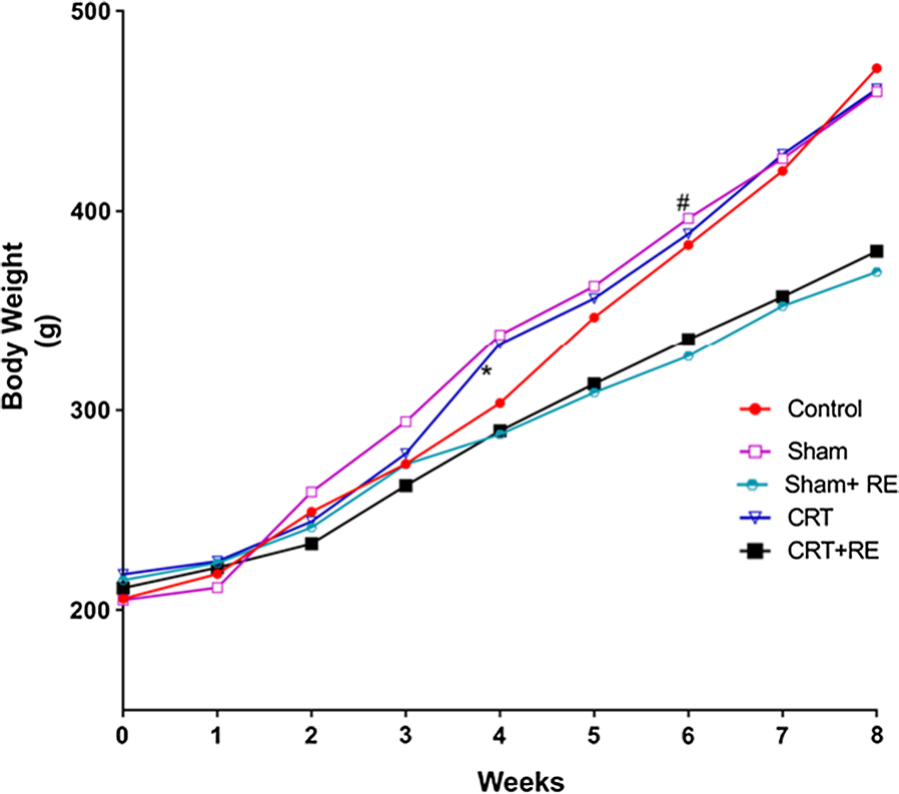
Weight of rats in each group at 8 weeks. Values are expressed as mean ± standard deviation (n = 10 per group). Control groups; sham groups; Sham + RE groups; CRT groups; CRT + RE, one-factor analysis of variance, Four weeks to eight weeks ^#^P < 0.01, versus Sham + RE groups; *P < 0.01, versus CRT + RE

No significant differences in body weight were observed amongst the groups from week 0 to week 3 (P > 0.05). By week 4, the rats in the Sham + RE group had a significantly lower body weight than the rats in the Sham group (P < 0.05). Moreover, the rats in the CRT + RE group had a significantly lower body weight than those in the CRT group (P < 0.05). From week 5 to week 8, the body weight of the rats in the Sham + RE and CRT + RE groups gradually decreased compared with that of the rats in the control, Sham and CRT groups; the difference was statistically significant (P < 0.01; Table 2). The body weight of the rats increased as time progressed, but their weight was reduced by the anti-resistance exercise training, thereby confirming the effectiveness of the exercise training program (Fig. 2).

**Table 2:** Weight of rats in each group from week 5 to week 8

Time (weeks)	Control (n = 10,g)	Sham (n = 10,g)	SJSham + RE (n = 10,g)	CRT (n = 10,g)	CRT + RE (n = 10,g)
The fifth	346.90 ± 36.27**##	362.50 ± 32.51*###	308.80 ± 27.45	356.40 ± 39.52#	313.10 ± 12.75
The sixth	383.20 ± 38.11**##	396.50 ± 35.57*###	327.10 ± 26.18	388.70 ± 41.70#	335.80 ± 7.74
The seventh	420.20 ± 32.61**##	426.30 ± 35.51*###	352.60 ± 23.62	428.40 ± 43.01#	357.30 ± 11.03
The eighth	471.50 ± 33.90**##	459.8 ± 36.32*###	369.70 ± 26.74	461.20 ± 36.84#	380.00 ± 10.81

Values are expressed as mean ± standard deviation (n = 10 per group) Control groups; sham groups; Sham + RE groups; CRT groups; CRT + RE, One factor analysis of variance

**P < 0.01, versus Sham + RE, ##P < 0.01, versus CRT + R, ###P < 0.01 CRT + RE, *P < 0.01, versus Sham + RE, #P < 0.01 versus CRT + RE

### CRT Formation in Rats

**Fig. 3:**
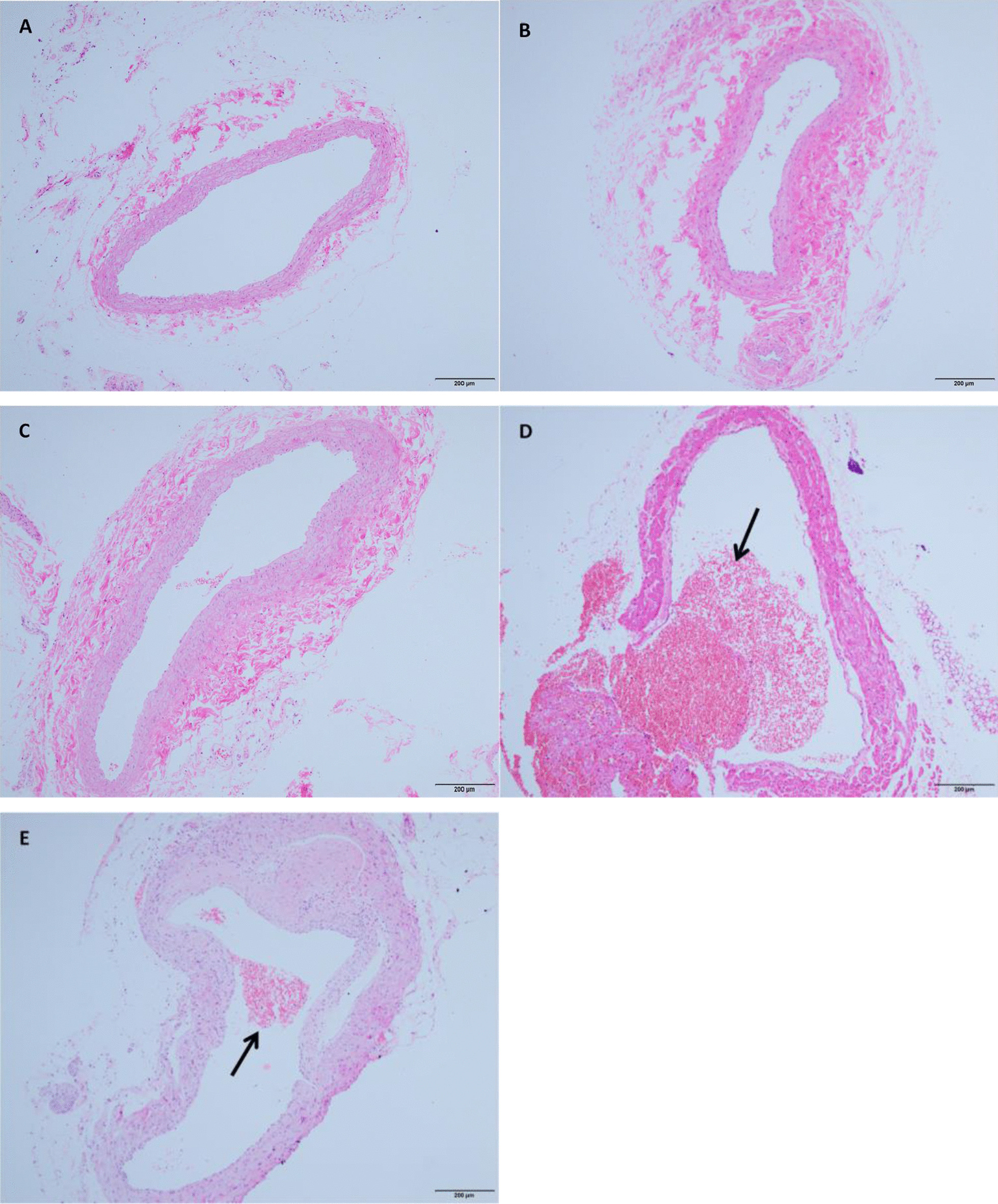
CRT Formation in Rats. **a** Control groups; **b** Sham groups; **c** Sham + RE groups; **d** CRT groups; **e** CRT + RE groups. The black arrow represents the CRT

The success rate of the indwelling catheter was 100%, and the catheter did not fall off during indwelling. All rats survived. No thrombosis was observed in the control, Sham and Sham + RE groups. In the CRT group, the success rate was 90% (9/10), whereas only a small amount of thrombosis with a formation rate of 30% (3/10) was observed in the CRT + RE group. The number of thrombosis cases in the CRT + RE group decreased compared with that in the CRT group, and the difference was statistically significant (P < 0.05; Fig. 3). In the CRT group, a large number of red blood cells, white blood cells and platelet beams were observed.

### Levels of oxidative stress in rats

**Fig.4:**
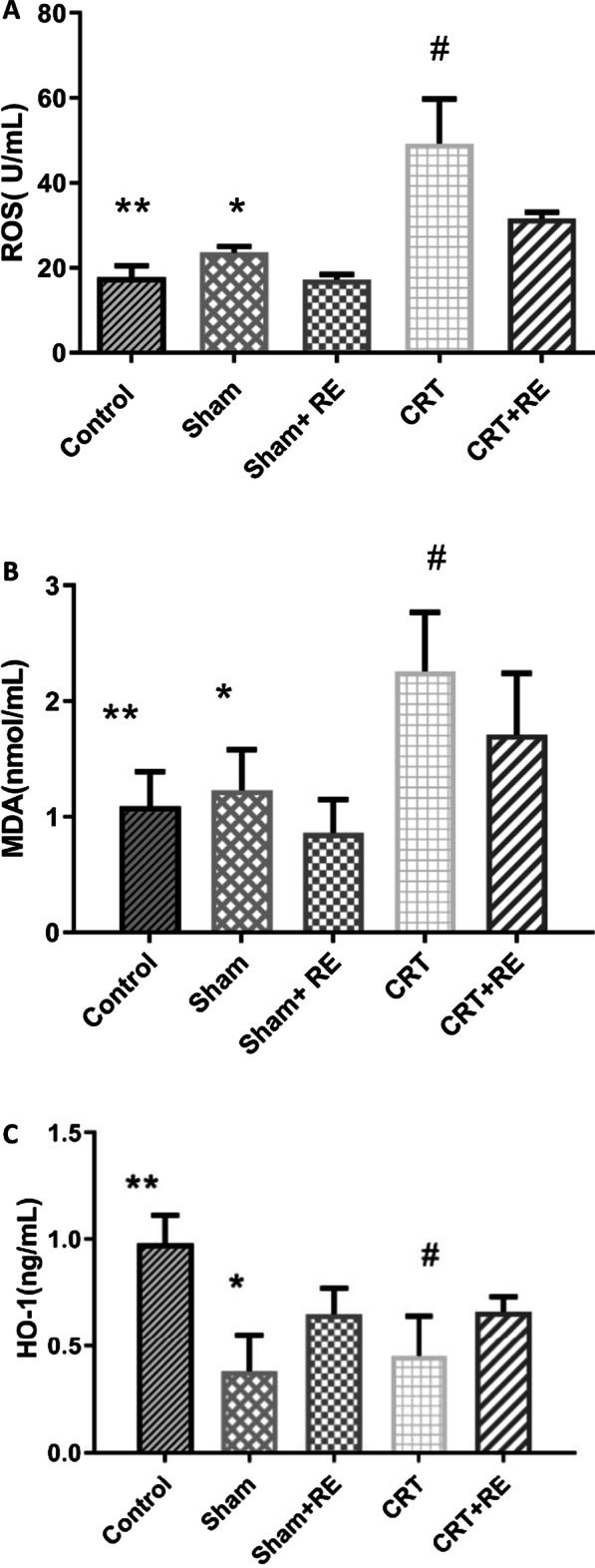
Levels of oxidative stress markers in the sera of rats. **a** ROS, **b** MDA and **c** HO-1. **P < 0.01, versus CRT, *P > **0.05**, versus Sham + RE, ^#^P < 0.01, versus CRT + RE

Compared with the control group, the oxidative stress levels of ROS and MDA in the CRT group significantly increased (P < 0.01), whereas that of HO-1 significantly decreased (P < 0.01). Compared with the CRT group, the oxidative stress levels of ROS and MDA in the CRT + RE group significantly decreased, whereas that of HO-1 significantly increased (Fig. 4). Compared with the Sham group, the oxidative stress level of Sham + RE group was not statistically significant (P > 0.05).

### Expression levels of miR -92a-3p and HO-1 mRNA in rat venous tissues

**Fig. 5:**
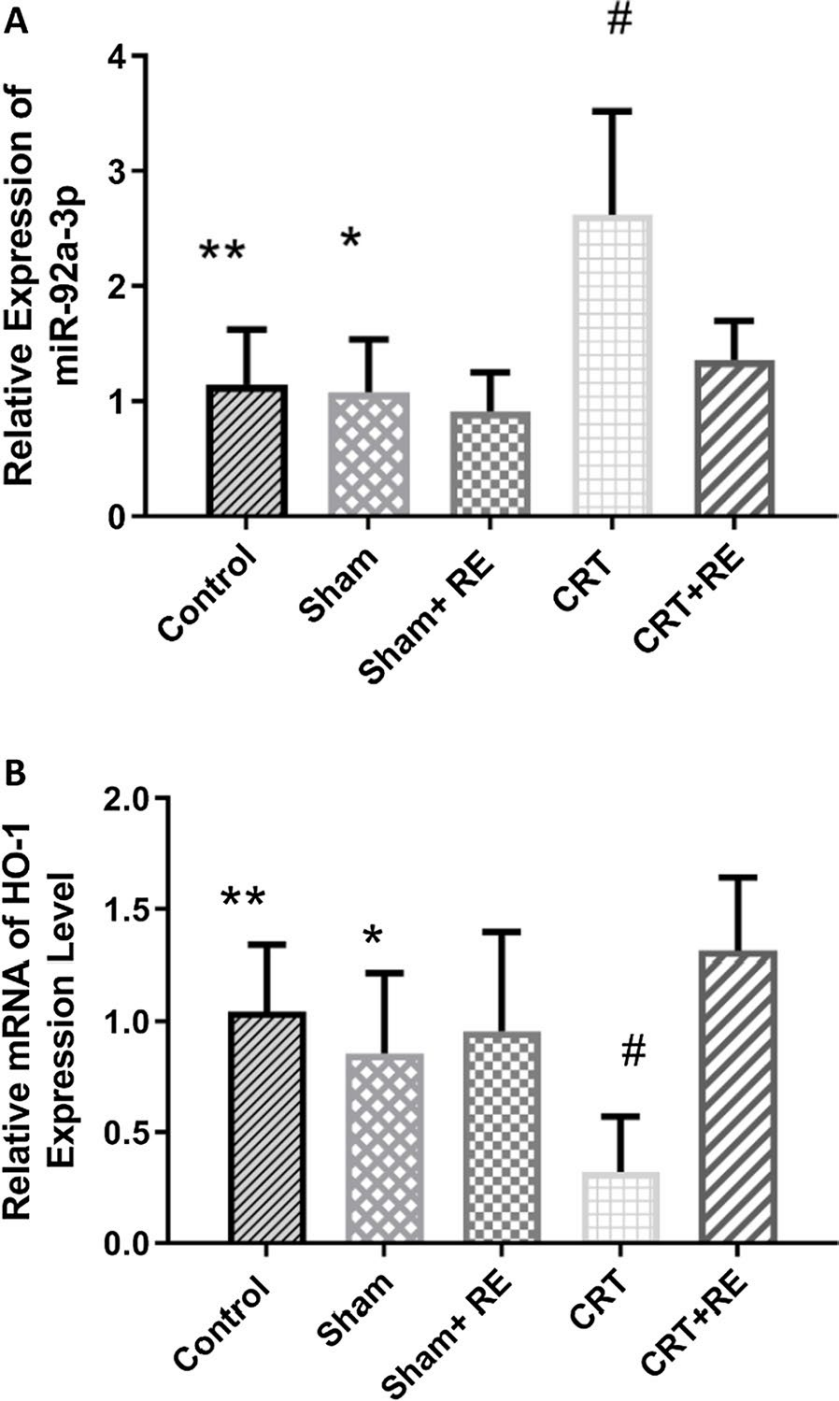
Expression of miR-92a-3p and HO-1 mRNA in rat tissues. **a** miR-92a-3p, **b** HO-1. The expression of miR-92a-3p increased in the venous tissue of rats in the CRT group but decreased in the CRT + RE group; the expression of HO-1 decreased in the venous tissue of rats in the CRT group but increased in the CRT + RE group.**p < 0.01, versus CRT groups, *P > 0.05, versus Sham + RE, ^#^p < 0.01, versus CRT + RE groups

The expression levels of miR-92a-3p and HO-1 mRNA in rat tissues were detected via RT-PCR. Their expression levels in the CRT group were significantly different from those in the control group (P < 0.01); in the CRT group, the expression of miR-92a-3p significantly increased, whilst the expression of HO-1 significantly decreased. Moreover, differences in miR-92a-3p and HO-1 mRNA expression between the CRT and CRT + RE groups were statistically significant (P < 0.01; Fig. 5). In the CRT + RE group, the expression of miR-92a-3p decreased, but the expression of HO-1 increased. However, no statistically significant difference was observed between the Sham and Sham + RE groups (P > 0.05).

### Correlation analysis between miR-92a-3p and HO-1

MiR-92a-3p was negatively correlated with HO-1 in rat venous tissues (r =  − 0.4197, P < 0.01).

### Expression of HO-1 and MAPK/NF-κB pathway proteins

**Fig. 6:**
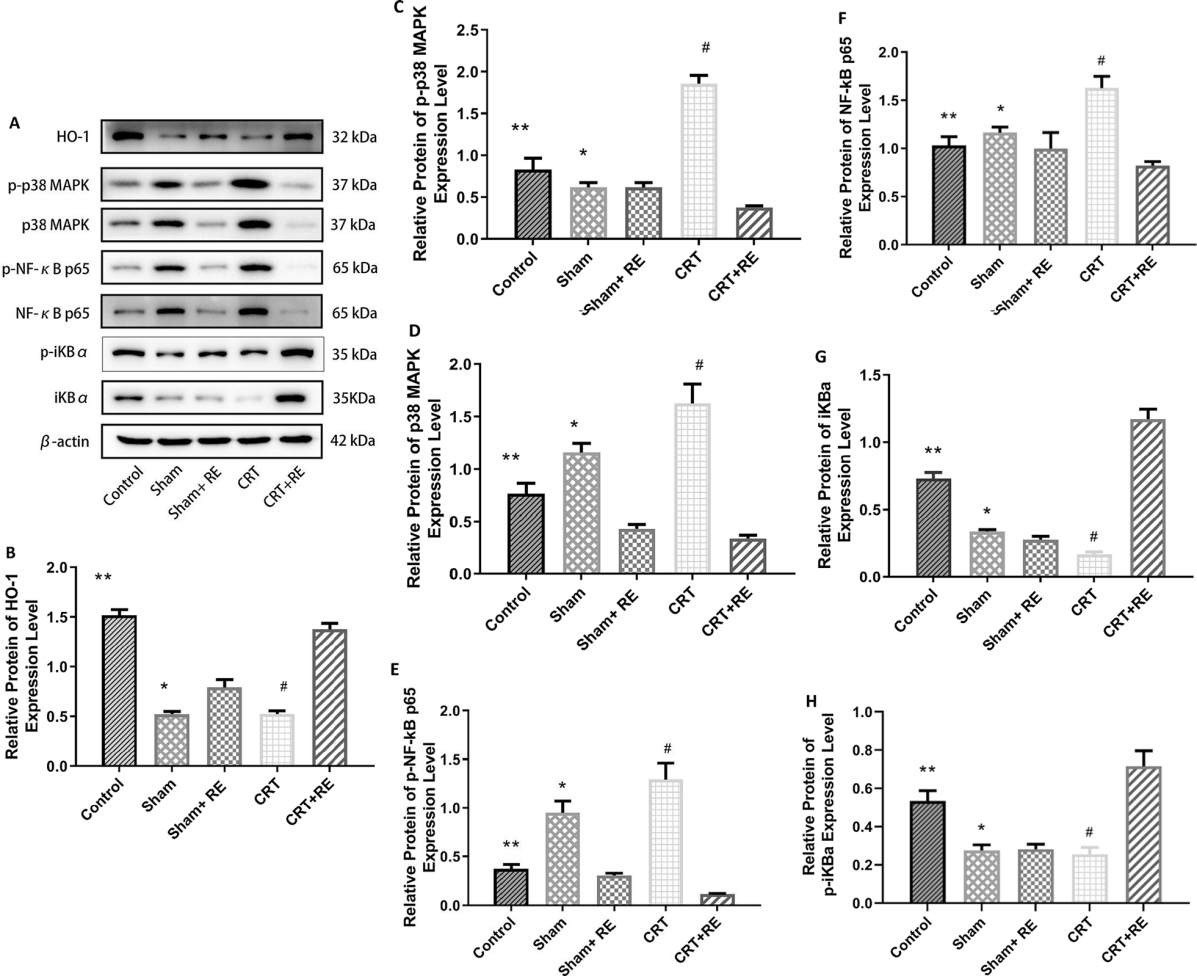
Expression of HO-1 and related proteins of the MAPK/ NF-κB pathway. **a** Western blot image of HO-1, p-p38MAPK, p38MAPK, P-NF-κB p65, NF-κB p65, P-IkBa and IkBa. **b**–**h** Analysis of protein expression of HO-1, p-p38MAPK, p38MAPK, P-NF-κB p65, NF-κB p65, P-IkBa and IκBa. **p < 0.01, versus CRT groups; ^#^p < 0.01, versus CRT + RE groups. **b**–**h** HO-1, p38MAPK, p-p38 MAPK and p-NF-kB p65,*p < 0.01, versus Sham + RE groups

On the basis of the results of Western blot analysis, compared with the control group, the expression of HO-1 in the CRT group decreased, the phosphorylation level of P38MAPK and phosphorylation P38 significantly increased, the phosphorylation levels of NF-κB p65 and NF-κB P65 significantly increased, p-IκBa and IκBa expression significantly decreased. Moreover, differences in the phosphorylation levels of HO-1, P38MAPK, phosphorylation p38 and NF-κB P65 between the Sham and Sham + RE groups were statistically significant, whereas differences in the levels of NF-κB P65,p-IκBa and IκBa were similar but not statistically significant. Compared with the CRT group, the expression level of HO-1 in the CRT + RE group increased, the phosphorylation levels of p38MAPK and phosphorylated P38 significantly decreased, the phosphorylation levels of NF-κB p65 and NF-κB P65 significantly decreased and the expression level of p-IκBa and IκBa significantly increased in the CRT + RE group (Fig. 6).

## Discussion

The incidence of thrombosis is the highest amongst all diseases, and its incidence rate is increasing. Through resistance exercise intervention, this study investigated the mechanism by which oxidative stress and miR-92a-3p prevent CRT in rats. Results showed that blood flow direction and velocity changed after catheter implantation, resulting in thrombosis formation, increased ROS production and MDA activity, decreased HO-1 level and increased miR-92a-3p expression. CRT also increased the release of pro-inflammatory cytokines, activated the P38MAPK/NF-κB P65 pathway and inhibited IκBa expression. Oxidative stress refers to excess ROS produced by the body when it is adversely stimulated, resulting in an imbalance between oxidative and antioxidant systems, which in turn leads to the accumulation of free radicals in cells and cell damage [27]. ROS content is an important marker of oxidative stress injury and one of the important factors leading to thrombosis occurrence and development [28]. In the cardiovascular system, ROS plays a role in controlling inflammation, proliferation, apoptosis, endothelial function and angiogenesis [29]. Aizawa et al. [28] demonstrated that an increase in ROS production in endothelial cells can promote thrombosis formation, leading to endothelial cell dysfunction. High ROS contents can convert polyunsaturated fatty acids on the cell membrane and produce MDA, further causing oxidative stress-induced tissue damage, increasing membrane permeability and rupturing the double-layered structure of the membrane.

Most miRNAs are effective therapeutic targets for cancer, but their roles in the cardiovascular system are not entirely clear [30]. As a member of the miR-17-92 family (i.e. miR-17, -18a, -19a/B, -20A and miR -92a), miR-92a-3p is highly expressed in vascular endothelial cells and participates in the regulation of vascular endothelial functions [31–33]. A sharp decrease in blood flow shear stress after catheter placement in the external jugular vein leads to the up-regulation of miR-92a-3p expression, vascular endothelial cell proliferation, apoptosis and thrombogenic molecular secretion and CRT formation [34]. As a member of the HO protein family, HO-1 is a common mammalian induction enzyme that degrades oxyhaemoglobin and produces antioxidants to protect cells from oxidative stress damage [35].

Pro-apoptotic pathways (e.g. MAPKs) and inflammatory response pathways (e.g. NF-κB) are important in regulating apoptosis and tissue damage. MAPKs and NF-κB are sensitive ROS signalling pathways. A sustained increase in ROS production can promote endothelial cell apoptosis and activate inflammatory responses [20]. NF-κB is maintained in its inactive form in the cytoplasm by binding to an inhibitory protein of the IκB family. In turn, this protein is phosphorylated and degraded under inflammatory stimuli, allowing the transfer of NF-κB p65 subunit phosphorylation and NF-κB dimer to the nucleus [36]. Punctures or long-term catheter placement in the vascular wall can cause oxidative stress injury in vascular endothelial cells. Moreover, they affect the biological functions of endothelial cells, suggesting that CRT and OS injury are closely linked to each other, which is consistent with the CRT model in the present study. Numerous studies confirmed that upper limb exercises can effectively promote blood circulation and reduce CRT occurrence [37]. In the current study, the resistance exercise intervention conducted for 8 weeks reduced ROS production and MDA activity, increased HO-1 level and down-regulated miR-92a-3p expression. Furthermore, the intervention reduced the P38MAPK/NF-κB P65 pathway and their phosphorylation levels and activated IκBa expression.

Thrombosis is a disease that involves numerous factors and systems. Histological features of thrombosis were explored in this study. In the control, Sham and Sham + RE groups, smooth and intact venous endothelial cells were observed under an optical microscope, and no thrombosis was found in the vascular lumen. Small amounts of powder were observed in the lumen of the rats in the Sham + RE group, but no thrombosis was observed. In the CRT group, inflammatory cells infiltrated around the vessel wall and formed a thrombus, and no endothelial cells were present at the adhesion site. In the CRT + RE group, a small number of red blood cells, white blood cells and platelet beams gathered in the lumen, and the severity of thrombus was less than that in the CRT group. The CRT in the CRT group was observed at the edge of the catheter. Trabecular platelets, red blood cells and white blood cells were found in the thrombus. Thus, clinical treatment of CRT remains a difficult task for clinicians. Anticoagulants [38], catheter surface coating [39], compression therapy [40] and grip strength training are commonly used in clinical settings [4]. Resistance exercise, also known as resistance training or strength training, usually refers to the process by which the body overcomes resistance to achieve muscle growth and increased strength [8]. Clinical grip strength training is a kind of resistance exercise training. Resistance exercises up-regulate the antioxidant defence system, decrease the concentration of cellular ROS and confer protection against oxidative stress-related diseases. The present study also proved that anti-resistance exercises changed blood flow shear stress, inhibited endothelial cells to repair oxidative stress and reduced ROS production, thereby improving the function of vascular endothelium and, to a certain extent, playing a role in preventing venous thrombosis [41, 42]. These results were consistent with the findings of Quinteiro [43].

By monitoring changes in miRNA expression, researchers found that miRNA circulation also changes as the intensity of resistance exercises increases [44, 45]. The current study showed that the resistance exercise intervention down-regulated miR-92a-3p expression, affected endothelial cell expression, repaired oxidative stress damage and inhibited CRT formation. Enhancing HO-1 activity by inhibiting the action of miR-92a can reduce oxidative stress damage and improve endothelial functions [34]. Intravenous up-regulation of miR-92a induces oxidative stress in endothelial cells, leading to endothelial inflammation and dysfunction [29]. We speculated that miR-92a-3p and HO-1 have a certain correlation with CRT. The results showed that miR-92a-3p was positively correlated with HO-1. However, the target of HO-1 regulation of thrombosis was not comprehensively explored. Therefore, the supposition that resistance exercise may mediate the targeting of HMOX1 by miR-92a-3p to regulate oxidative stress and prevent CRT occurrence warrants further experimental verification.

Shear stress is also a strong inducer of HMOX1 that can inhibit leukocyte adhesion and platelet aggregation. HO-1 can inhibit the formation of venous thrombosis by alleviating oxidative stress mechanisms [46]. However, the role of HO-1 in reducing tissue damage, especially in venous thrombosis, through its antioxidant activity is poorly understood. Recent studies explored potential therapeutic tools for manipulating apoptosis, inflammation and oxidative stress to improve the outcome of vascular diseases. Studies of the HO-1 promoter region revealed that the combination of the presence of transcriptional response elements, including activator protein I, activator protein II, NF-κB and interleukin-6 response elements, with anti-oxidant response elements, induces the inhibition of the proliferation of vascular smooth muscle cells [47]. Aside from the fact that HO-1 expression is induced by oxidative stress, HO-1 also plays an important cellular protective role in various inflammatory diseases. Previous studies explored the mechanism of oxidative stress in rat brain as a function of age. Western blot and immunohistochemistry analyses revealed that the expression of the HO-1 protein in the heart antioxidant enzyme slightly decreased [48], whereas that in the kidney slightly increased. However, HO-1 expression in the kidney substantially decreased by the fifth week [49]. Therefore, HO-1 expression is slightly different in varying tissues and at various time points. Nevertheless, HO-1 was undeniably structurally up-regulated amongst the top climbers, and the high HO-1 expression level was maintained months after reaching the peak [50]. Eight weeks of climbing resistance training induced an increase in HO-1 expression, which led to an antioxidant reaction in the blood vessels and initiated the corresponding protective mechanism.

### Limitations and prospects

This study provides a theoretical basis for CRT prevention via resistance exercise intervention. This process is important in future translational research, especially for patients in ICUs or with cancer undergoing chemotherapy. Nevertheless, this study has several limitations. Firstly, thrombosis was only assessed by pathology. If catheterisation and CRT monitoring can be combined with ultrasound technology, thrombosis formation and the relationship between blood flow changes and CRT can be determined in various forms. Secondly, platelet activation was ruled out; however, the clotting system may be involved in CRT formation. We monitored thrombus formation at different time periods spanning 14 days and at the end of 2 months [22]. We will continue examining changes in thrombus formation at different time periods for 1 month under resistance exercise intervention. Moreover, we will constantly track the effects of changes in resistance exercise training at different time points on HO-1 and promoter regions, especially on the protection and improvement of blood vessels.

## Conclusion

Vascular catheterisation resulted in endothelial cell oxidative stress damage and miR-92a-3p-mediated inflammation, ultimately leading to thrombosis. Resistance exercise intervention accelerated blood flow speed, repaired oxidative stress damage, reduced ROS production and decreased CRT incidence, thereby improving cardiovascular functions. Hence, resistance exercises are important in the prevention and treatment of CRT. Resistance exercise may mediate the regulation of oxidative stress by targeting HMOX1 via miR-92a-3p to prevent CRT occurrence. However, this supposition warrants further research. Resistance exercise intervention provides a strong theoretical basis and research direction for the prevention and treatment of CRT.

## Supplementary Information

**Additional file 1** The Tab of Animal Experimental Ethical Inspection.

## Data Availability

The datasets used and/or analysed during the current study are available from the corresponding author upon reasonable request.

## Abbreviations

CVC: Central venous catheter
CRT: Catheter-related thrombosis
ROS: Reactive oxygen species
MDA: Malondialdehyde
HO-1: Heme oxygenase-1
miR-92a-3p: MicroRNA-92a-3p
P38 MAPK: P38-mitogen-activated protein kinase
NF-κB: Nuclear factor kappa-B
ELISA: Enzyme-linked immunosorbent assay
qPCR: Quantitative polymerase chain reaction
