# Supplementary material for: Resistance exercise affects catheter-related thrombosis in rats through miR-92a-3p, oxidative stress and the MAPK/NF-κB pathway
Source: BMC Cardiovasc Disord. 2021 Sep 16;21:440. doi: 10.1186/s12872-021-02233-w (PMC8444419; doi:10.1186/s12872-021-02233-w)
Supplement: Supplementary file 1 — Additional file 1 The Tab of Animal Experimental Ethical Inspection. [file 12872_2021_2233_MOESM1_ESM.docx]

| HO-1 |  | 32 KDa |
| --- | --- | --- |
| p-p38 MAPK |  | 37 KDa |
| p38 MAPK |  | 37 KDa |
| p-NF-kappa p65 |  | 65 KDa |
| NF- kappa B p65 |  | 65 KDa |
| iKB alpha |  | 35 KDa |
| β-actin |  | 42 KDa |
